# Systematic review of performance-enhancing health worker supervision approaches in low- and middle-income countries

**DOI:** 10.1186/s12960-021-00692-y

**Published:** 2022-01-06

**Authors:** Rachel Deussom, Doris Mwarey, Mekdelawit Bayu, Sarah S. Abdullah, Rachel Marcus

**Affiliations:** 1 HRH2030 Program, Chemonics International, 1717 H Street NW, Washington, DC 20006 United States of America; 2 Human Resources for Health Consultant, Formerly With Chemonics International, PO Box 26432–00100, Nairobi, Kenya; 3 Office of Health Systems, Bureau for Global Health, United States Agency for International Development, 1300 Pennsylvania Avenue NW, Washington, DC 20004 United States of America

**Keywords:** Human resources for health, Health workforce, Workforce development, Supportive supervision, Health systems, Performance management, Enhanced supervision, Systematic review, Quality improvement

## Abstract

**Background:**

The strength of a health system—and ultimately the health of a population—depends to a large degree on health worker performance. However, insufficient support to build, manage and optimize human resources for health (HRH) in low- and middle-income countries (LMICs) results in inadequate health workforce performance, perpetuating health inequities and low-quality health services.

**Methods:**

The USAID-funded Human Resources for Health in 2030 Program (HRH2030) conducted a systematic review of studies documenting supervision enhancements and approaches that improved health worker performance to highlight components associated with these interventions’ effectiveness. Structured by a conceptual framework to classify the inputs, processes, and results, the review assessed 57 supervision studies since 2010 in approximately 29 LMICs.

**Results:**

Of the successful supervision approaches described in the 57 studies reviewed, 44 were externally funded pilots, which is a limitation. Thirty focused on community health worker (CHW) programs. Health worker supervision was informed by health system data for 38 approaches (67%) and 22 approaches used continuous quality improvement (QI) (39%). Many successful approaches integrated digital supervision technologies (e.g., SmartPhones, mHealth applications) to support existing data systems and complement other health system activities. Few studies were adapted, scaled, or sustained, limiting reports of cost-effectiveness or impact.

**Conclusion:**

Building on results from the review, to increase health worker supervision effectiveness we recommend to: integrate evidence-based, QI tools and processes; integrate digital supervision data into supervision processes; increase use of health system information and performance data when planning supervision visits to prioritize lowest-performing areas; scale and replicate successful models across service delivery areas and geographies; expand and institutionalize supervision to reach, prepare, protect, and support frontline health workers, especially during health emergencies; transition and sustain supervision efforts with domestic human and financial resources, including communities, for holistic workforce support. In conclusion, effective health worker supervision is informed by health system data, uses continuous quality improvement (QI), and employs digital technologies integrated into other health system activities and existing data systems to enable a whole system approach. Effective supervision enhancements and innovations should be better integrated, scaled, and sustained within existing systems to improve access to quality health care.

**Supplementary Information:**

The online version contains supplementary material available at 10.1186/s12960-021-00692-y.

## Background

Health systems largely depend on health worker performance to provide health for all [1, 2]. However, insufficient support to build, manage and optimize human resources for health (HRH) results in an insufficient quantity of health care workers (HCWs) in low- and middle-income countries (LMICs), which in turn perpetuates health inequities and produces low-quality health services [3, 4].

HCW supervision is intended to improve the quality and coverage of health services. However, its effectiveness is dependent on the context, availability of other health systems inputs, implementation factors, and the means of and level at which supervision is evaluated[5]. Bailey et al.’s [6] systematic review of health worker supervision concluded it “[did] not find a solid foundation on which to base clear conclusions on the effect of supportive supervision on quality of care or clinical outcomes; supportive supervision alone, however, does not seem to be effective in improving quality of care in contexts, where the required health system inputs are inadequate.” Supervision program parameters are often ill defined, and there is limited evidence on the direct attribution of supervision beyond HRH performance, motivation and satisfaction, to broader clinical and health outcomes.

Per Rowe et al. [5] components of supervision programs vary greatly depending on the country and scale of implementation, health focus area, funding source, and availability of other health systems inputs. The large HCPPR database reviewed five decades of literature to assess the HCW supervision and its effectiveness—whether positive or negative—when combined with a range of other performance interventions. However, for country governments and practitioners seeking evidence and best practices on how to implement effective HCW supervision, there is limited elaboration and discussion of the practical inputs and processes of effective supervision systems, particularly as emerging health threats further constrain health systems and HCW support is needed more than ever. In addition, as many countries seek to expand the reach of primary health care services through community health programming, there is heightened need to ensure that all health workers are adequately supported and supervised to deliver high-quality care.

Our systematic review sought to identify and analyze the components of recently implemented interventions designed to strengthen HCW supervision in LMICs that demonstrated improved health workforce performance or other positive health system effects. We then conducted a structured analysis of the identified interventions to describe the inputs and processes underpinning each successful supervision approach.

## Methods

The USAID-funded Human Resources for Health in 2030 (HRH2030) Program undertook a database search of white and grey literature to gather evidence on supervision approaches using defined terms and inclusion criteria. The first-round search was conducted in June 2018 and updated in October 2020. Our information sources included randomized controlled trials, quasi-experimental studies, scoping reviews, end-of-project reports, systematic reviews, qualitative studies, journal articles, country case study reports, technical briefs, and conference presentations, among others (Table 1).

**Table 1:** Enhanced supervision search results disaggregated by database

Database	Initial search results	After removing repeats	Related to health (for multi-disciplinary databases)	Since 2010	In English	Further search using database filters	Relevance of title & abstract
Cochrane Database of systematic reviews	313	309	281	226	226	226	3
Global Health: Science & Practice journal	312	144	33	27	27	27	32
GlobalHealth & PubMed	222	222	170	103	103	103	33
Health Care Provider Performance Review (HCPPR)^b^	118	118	12	3	3	3	3
Health Systems Evidence	543	531	156	287	287	287	1
Healthcare Management Information Consortium (HMIC)^a^	0	0	0	0	0	0	0
HRH Global Resource Centre^a^	94	94	15	79	79	79	6
mHealth Compendium Databases^a^	80	16	16	16	16	16	4
Popline	49,873	49,873	118	118	118	118	3
References from Bailey et al. [6]^a^	10	10	10	10	10	10	10
ResearchGate	133	132	132	110	108	108	8
The Lancet	40	36	13	9	9	9	4
USAID DEC	15,488	15,439	4052	2972	2972	364	20
WHO COVID Database^b^	54	51	1	0	0	0	0
WHO Global Health Library^a^	613	383	383	362	362	362	0
Total	67,893	67,358	5392	4322	4320	1712	127

^a^Retired or unavailable databases that were only part of the first-round search in June 2018

^b^New databases that were only part of the second-round search in October 2020

**Fig. 1:**
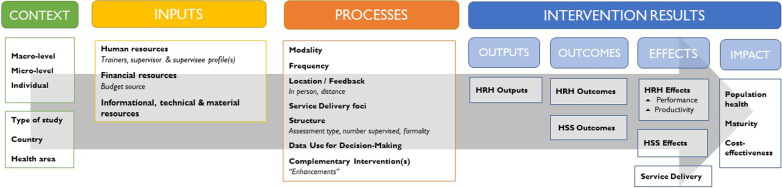
Conceptual framework for reviewing enhanced supervision approaches (Source: HRH2030 2019. Adapted from GHWA [8], Dieleman et al. [7], and informed by Campbell et al. [3])

For analysis, we adapted the conceptual framework developed by Dieleman et al. [7] to build on previously defined dimensions of HCW performance, adding in performance indicators used by the World Health Organization’s Global Health Workforce Alliance [8] (Fig. 1)*.*

Articles meeting our review criteria were examined to ensure their relevance to health worker supervision and were assessed for the quality of their methodology using Critical Appraisal Skills Program (CASP) checklists for the studies relevant to the available checklists. A team of four reviewers identified, classified, and analyzed a total of 57 articles using an Excel-based template of the framework, noting emerging themes and patterns across different settings, health worker types, program goals, modalities, pedagogies, other enhancements, and complementary interventions. We analyzed each intervention to qualify and summarize the inputs, processes, and results of the supervision approach and then classified them using the framework categories. Framework categories to classify approaches were modified as new themes emerged during analysis. We then conducted a quantitative analysis to determine the frequencies for each category. Finally, to develop our main recommendations for enhancing supervision, we conducted a qualitative analysis to extrapolate the most critical and/or promising supervision enhancements—highlighting key components, noteworthy results, and scaled or sustained approaches.

## Results

### Study selection

**Fig. 2:**
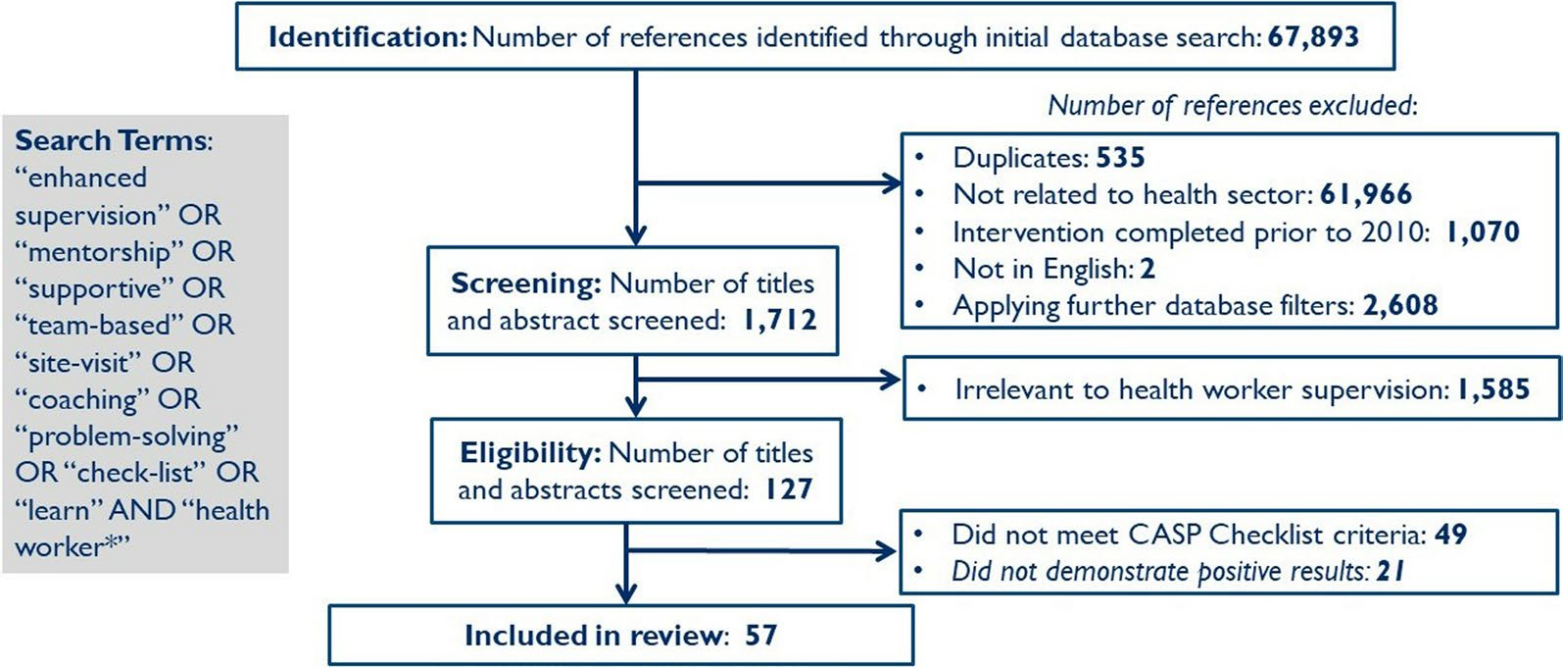
Enhanced supervision review: database search results

The first- and second-round database searches yielded a total of 67 893 articles, of which 1727 met our primary criteria, including health sector relevance and publication in English in 2010 or later. The titles and abstracts of these publications were then reviewed further for relevance, yielding 127 articles. We then applied the CASP checklists and inclusion criteria of demonstrated positive results, yielding a total of 57 studies for the review (Fig. 2; Additional file 1).

### Analysis of HCW supervision enhancements

**Fig. 3:**
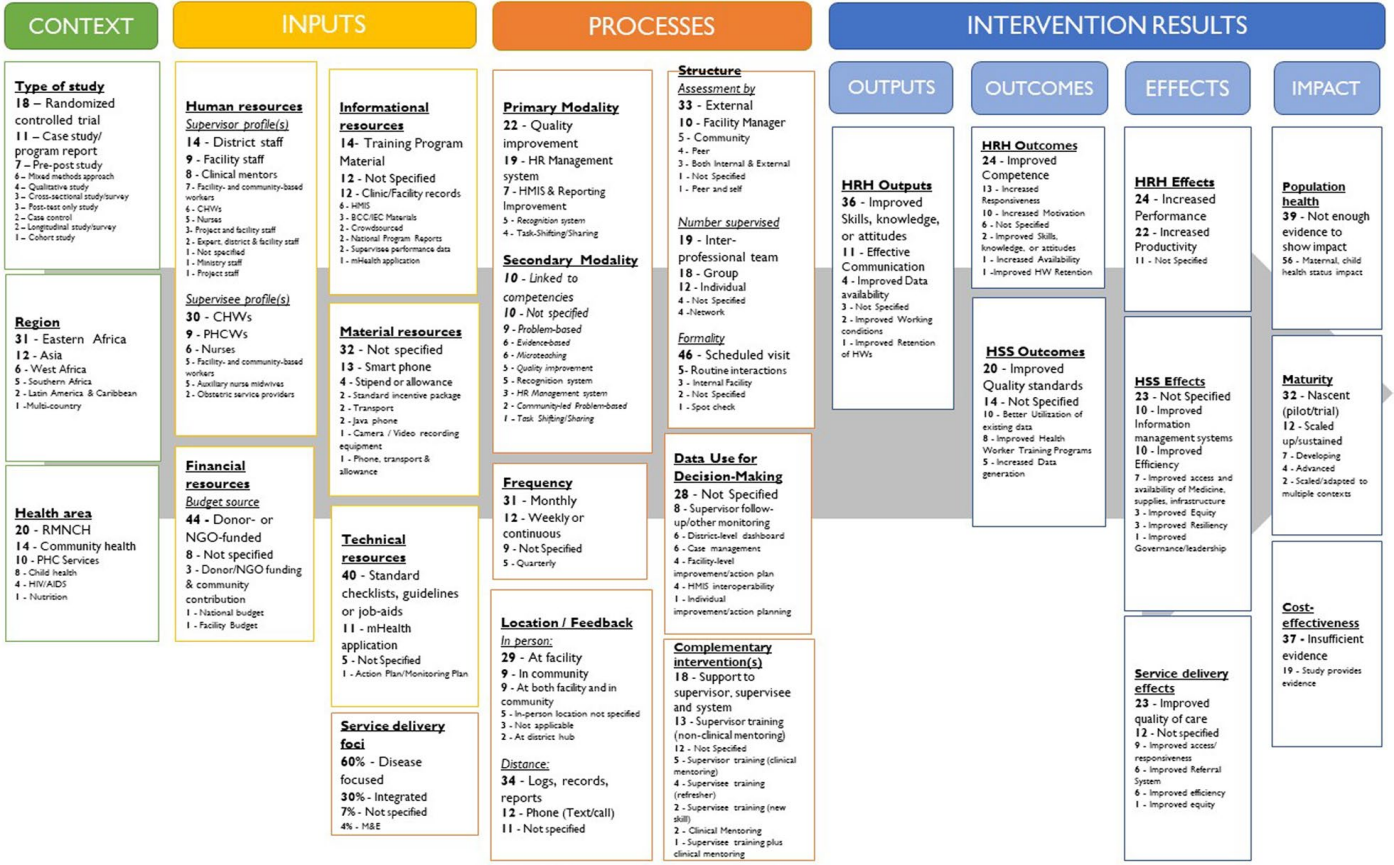
Quantitative summary of enhanced supervision approaches reviewed using conceptual framework (*n* = 57) (Source: HRH2030, updated 2021. Adapted from GHWA [8], Dieleman et al. [7], and informed by Campbell et al. [3])

We present the quantitative and qualitative findings of our review of successful HCW supervision interventions according to the conceptual framework areas: context, inputs, processes, and intervention results (Fig. 3).

### Context

Per our quantitative analysis, studies reviewed included 18 randomized controlled trials (32%), 11 case studies (19%), seven pre–post-tests (12%), and six mixed methods (11%). Forty-two of the studies documented approaches in Africa (74%), 12 in Asia (21%), and two in Latin America & the Caribbean (4%). Twenty studies focused on reproductive, maternal, newborn and child health (35%), 14 on community health (25%), ten on primary health care (18%), eight in child health (14%), four HIV/AIDS (7%), and one on nutrition (2%).

Per our qualitative analysis, the studies reviewed varied in focus and context. Many identified macro-level determinants of HCWs’ baseline performance and related to the overall health system, socio-economic and political context, education system, and the labor market. Many supervision approaches in these studies were driven by a new national health sector policy, guideline, or training program, many of which focused on the professionalization or increased responsibility assigned to community health workers (CHWs). In addition, studies examined micro-level factors affecting HCW performance: workplace dynamics, individual attributes of health workers, high workloads, high turnover, absenteeism, inefficient processes, vast geographic distances, limited equipment and supplies, and limited community trust and health service utilization, especially for CHWs [9, 10].

### Inputs

Supervision inputs we reviewed included human, financial, informational, material, and technical resources. For human inputs (e.g., supervisor and supervisee profiles), 14 studies observed supervision with district staff as the supervisors (25%), nine with facility staff supervisors (16%), and six with CHWs as supervisors (11%); 28 were unspecified. Thirty studies (53%) observed CHWs as the supervisees, with others focused on primary health care staff supervision, notably nurses. Forty-four supervisory interventions (77%) were funded by non-governmental organizations (NGO) or donors; eight did not specify funding sources (14%), three were cost-shared (5%), one was funded by the facility (2%), and one by the national budget (2%). Of the 38 approaches, where supervisors used informational resources to prepare supervision, 14 approaches reviewed used training materials and 12 used facility-level records (21%), while 12 others (21%) were unspecified. For five approaches, mHealth applications supported CHW-focused supervision with individual supervisee performance data or “crowdsourced” information from supervisees. Present in 40 approaches (70%), the most frequently cited technical resource inputs were standards of care checklists, guidelines, or health worker job aids. Smartphones and mHealth applications were key inputs for the supervision approach in 13 (23%) and 11 (19%) of studies reviewed, respectively.

### Processes

Supervision processes were analyzed in terms of inputs and processes by primary and secondary modality, frequency of supervision, location and delivery of feedback provided by the supervisor, structure of supervisor approach, as well as interventions complementary to supportive supervision—including enhancements for the supervisor, supervisee, and/or health system.

#### Primary and secondary modalities

We reviewed the primary and secondary modalities (i.e., methods or procedures) of the successful supervision approaches. Cited in 22 studies (39%), quality improvement (QI) was the most common primary modality for supervision, often enhanced with various secondary modalities. Nineteen studies (33%) documented a standard HR management approach, to supervision, though some of these interventions also employed a problem- or competency-based approach. Supervision interventions supporting task shifting/task sharing frequently used the QI modality, complemented by clinical mentoring. For example, applying monthly QI visits with clinical mentoring for nurses enhanced their scope and upgraded their skills for integrated management of adult and child illness and antenatal care services [11–13].

Of the 22 supervision approaches using QI as the primary modality, they contributed positive effects at all levels:

- Outputs: 21 approaches improved HRH skills, knowledge, and attitudes;
- Outcomes: 12 approaches improved HW competence, and seven approaches improved quality of standards of care;
- Effects: Six improved HRH performance and/or productivity and 12 improved the quality of care;
- Impact: Nine supported improved population health (compared to only three of the 19 HR management systems approaches).

Our review supports the effectiveness of QI evidenced by previous reviews [14–16].

Using existing health management information system (HMIS) data (e.g., service delivery indicators) to inform the supervision approach supported a range of different health system goals, including task shifting of mid-level providers in Uganda [17]; improved CHW performance and system efficiencies for nutrition services in India [18]; improved quality of care for private sector and/or community-based health providers in malaria and family planning services across Africa and Asia [19]; and improved referral systems for CHWs [20, 21]. More research is needed to connect the impact of HMIS-informed supervision approaches on service delivery effects. Four HMIS-focused supervision approaches demonstrated cost-effectiveness [22–25].

Combining supervisor training on clinical mentorship, plus supervisee clinical mentoring and supervision with a standardized job aid to support clinical decision-making helped 85% of ART patients initiate treatment with nurses in South Africa [26]. In Senegal, the combination of HCW performance support, mentoring, workplace improvements, and community support increased patients’ informed choice by 86% over 6 months [27]. In Ghana, professionalizing district managers, supervisors, and communities to take a “client-centered” approach to HRH management provided staff with an effective enabling environment [28].

#### Frequency

In 46 of the 57 approaches (81%), supervisory visits were scheduled. Of these 31 approaches scheduled monthly visits 12 scheduled weekly/continuous visits, and four scheduled quarterly visits. Twenty-three of the 31 approaches (74%) with monthly supervision visits showed increased HCW productivity and performance, compared to only one of the four (25%) that scheduled quarterly visits. More intensive, frequent, or continuous supervisory support was shown to be effective immediately after a new skill or task was imparted [29, 30]. However, the quality of supervision was cited as more important than its frequency for CHW supervision in Uganda [31].

#### Location and delivery of feedback

We examined how and where supervision feedback was shared with HCWs. High-quality, timely feedback was shown to benefit both supervisors and supervisees whether provided in-person (*n* = 11), at a distance (*n* = 3), or in combination (*n* = 43). All in-person feedback for supervisory visits occurred at the health workers’ place of work, in facilities or communities. For example, for CHWs supervised at a facility hub in Uganda, quarterly community-based supervision was combined with monthly on-site CHW meetings, contributing to their motivation and productivity [32]. In Ethiopia, Kenya, Malawi, Mozambique, and Tanzania, group CHW supervision at the facility combined with supervisor training served to improve motivation and the efficiency of supervisory processes [33, 34].

Of the 46 studies that assessed providing distance feedback, about 34 used existing records/reports, and 12 used phone/text messages. Distance feedback, including sharing summaries of service delivery data indicators, appeared to effectively complement in-person visits. Approaches with QI and HR management supervision modalities included feedback loops through sharing of reports, logs, and records. Whether texting or calling, phone communication was most frequently documented when the primary supervision modality was a recognition system [35, 36], and often documented more effective communication, increased health worker responsiveness, and increased data use [18, 37, 38]. Network-wide communications, such as WhatsApp group and peer-to-peer discussions supporting CHWs in Kenya, were considered favorable to reinforce standards of care and clinical guidance, provide activity updates, reinforce accountability through photo sharing, and recognize and motivate CHWs [36, 39].

#### Structure of supervision approaches

We reviewed the relationship of the supervisor to supervisee, the number of supervisees visited, and the level of formality of the visit (e.g., scheduled visit versus routine interaction). Of the 57 studies, 33 supervision approaches (58%) used external evaluators to assess performance (e.g., individual or team who was not part of facility or community, often from a district, NGO, or project). Ten approaches relied on facility managers (18%), five used community assessments (9%), four used peer assessments (7%) and one combined peer and self-assessments (2%). Approaches combining internal and external assessments demonstrated effective support to interprofessional teams [40, 41]. Thirty-six approaches used interprofessional or group assessments (63%), of which many were to supervise CHWs.

#### Data-use for decision-making

Twenty-eight (49%) studies did not specify *how* supervisory visit data, reports and other information were used *after* the visit to inform subsequent actions and intervention. For QI modalities, continual data review may have been considered implicit by their authors but was not specified [12–14, 41–47].

#### Complementary interventions

We reviewed studies that used supervision plus other interventions to address underlying health system challenges that may hinder HCW performance. “Whole of system” approaches providing complementary support to HCW enabling environments, cited in 25 studies (44%), were found to be effective across several areas, including improving health information systems, increasing process efficiencies, and providing better access to medicine, supplies, and health infrastructure. eighteen studies (32%) focused on the quality of supervision by seeking to improve supervisors’ HR management skills, some using the cascade model of clinical mentoring [12–14, 48]. Linking supervisee training to post-training supervision visits, whether for a new skill or a refresher training, was used in fewer instances. Task shifting-focused supervisory interventions generally followed HCW new skills training [17, 49].

### Intervention results

We explored the interventions studied to establish a range of results across four separate but related levels: outputs, outcomes, effects, and impact, summarized in Fig. 3.

#### Outputs

Fifty-four supervision approaches demonstrated improvements in HRH outputs (95%), of which 36 cited improved skills, knowledge, or attitudes. Some studies demonstrated results at several levels of Bloom’s taxonomy of learning outcomes [50], from testing individual HCW knowledge and comprehension of health areas or tasks [11, 51–53] to measuring the application of specific clinical tasks and adherence to standards of care [11–13, 26, 28, 54]. Improved attitudes documented by the studies included improved job satisfaction, commitment, and conscientiousness [33]; increased awareness of the importance of posting facility job aids [55]; increased recognition and support [31], and attitudes toward patients [45].

The second most frequent HRH output was effective communication, reporting, and information sharing. Eleven studies reported communication improvements (19%), not only for HCWs and supervisors but also for HCWs and clients and within facility teams. Outputs less frequently cited were improved data availability, improved working conditions, and improved retention of HCWs [37].

#### Outcomes

Most studies measured positive health workforce outcomes (e.g., improved availability, responsiveness, competence, or motivation) and HSS outcomes (e.g., improved quality standards, data use, service utilization, or workforce training programs). Twenty-four studies reported improved competence (42%) and twenty studies reported improved quality standards (35%). For CHW supervision studies, which focused primarily on RMNCH in India, Ethiopia, Kenya, Pakistan, and Tanzania, delegating supervisory roles to CHW supervisors or peers was shown to be effective, with results including improved CHW supervisee motivation [33, 35], safety [48], communication [39], and improved skills and standards of care [42].

When reviewing supervision modalities against outcomes, QI was the most frequently cited as improving health worker competence, with twenty studies, whereas HR management system modalities accounted reported improved motivation, cited in ten studies. Many studies discussed the importance of both constructive and positive feedback in improving HR management [52].

HSS outcomes related to data use—better utilization of existing data and increased data generation—were achieved through HMIS and reporting system modalities [22, 23, 43, 51, 56], as well as through HR management system improvements [19, 20, 28, 57], QI [41, 43, 58], recognition systems [35, 36], and task-shifting/sharing modalities [17].

In three instances, where external, donor-supported project staff supervised providers alongside local government facility staff, the local government subsequently sustained the approaches [23, 24, 55].

#### Effects

Most studies cited improvements at the effects level. Forty-six improved HRH performance and productivity (81%), 45 improved service delivery (e.g., responsiveness, quality of care, and referral systems) (79%), and 34 improved other health systems components (e.g., governance, financing, information, medicine, supplies, and infrastructure) (60%). Specifically, 24 improved health worker performance, 22 increased productivity, and 23 improved quality of care. In terms of commonly reported health systems effects, ten approaches improved information management systems, ten improved efficiency, and seven improved access to and availability of supplies, medicine, and infrastructure.

#### Impact

We explored the impact of supervision approaches in terms of population health, maturity, and cost-effectiveness. Eighteen studies (32%) attributed impact on maternal, newborn, and child health (MNCH) to supervision approaches [12, 13, 17, 21, 27, 36, 41, 42, 49, 52, 58–60]. Of these, four were reported to have been scaled up and sustained: the Safer Deliveries intervention in Zanzibar [20, 21], a community-led supervision intervention with traditional birth attendants in Ecuador [41], a digital health intervention for integrated family health services in Bihar, India [38], and the MESH-QI approach for supervision and task sharing support for nurses providing antenatal care in Rwanda [11–13, 60].

Thirty-two approaches documented in this review (56%) were at the nascent stage, or the lowest stage of maturity, and included pilots, trials, and other interventions not yet implemented at scale. Seven studies were at the developing stages (12%), and four were at advanced stages (7%).

Despite the successful results of all studies reviewed, only 12 studies (21%) reported that the supervision enhancements were sustained or scaled beyond the study period. Two approaches—MESH-QI and the Health Network Quality Improvement System (HNQIS)—were scaled up and adapted to multiple contexts [11–13, 19, 60]. MESH-QI has been implemented in Rwanda, Liberia, and Malawi. HNQIS has been applied in 19 countries across different health areas and within both the public and private sectors. To better understand these two approaches, we conducted in-depth interviews with program implementers and developed two qualitative case studies that are structured according to our conceptual framework [61].

Five approaches—the MESH-QI, Safer Deliveries, and Bihar digital heath interventions, as well as a continuous quality improvement intervention for CHWs in South Africa and mentoring approach in Senegal—demonstrated population health impact, scalability, and cost-effectiveness [11–13, 21, 38, 59, 60]. A CHW-focused digital health supervision approach for family planning in Tanzania also reported population-level impact and cost-effectiveness; however, it was a nascent intervention [36].

## Discussion and recommendations

The results from our review indicate that externally funded QI-based, digital, integrated HCW supervision enhancements demonstrate HCW performance and service delivery improvements across a range of health areas. However, there is very limited documentation of domestically funded, larger scale, longer term supervision enhancements that have been sustained over time with local investment. As many LMIC governments aim to institutionalize and sustain CHW programs, scalable, affordable supervision systems for community-based health workers become increasingly imperative.

A limitation of the study is that the indicated search criteria provided mainly peer-reviewed journals, and results were biased toward those studies with more substantial external funding. Additional documentation and research, including cost-effectiveness studies, should be conducted as nascent approaches are brought to scale, to enable thorough analysis of the modalities that can sustainably impact population health.

While beyond the scope of the review, we are aware that some approaches have been sustained and/or scaled. For example, MESH-QI remains a technical approach with comprehensive guidance that Partners in Health and country governments continue to implement [62]. Safer Deliveries supervision approaches have been implemented and scaled across Zanzibar, Tanzania, with continued collaboration between D-tree International and the Zanzibar Ministry of Health [63].

Stewardship for implementing, evaluating, and financing effective HCW supervision approaches must further shift from external partners to national and subnational governments. To address the dearth of country-led peer-reviewed literature on HCW supervision, ministries of health should engage with local research partners, schools of public health, and national health institutes to strengthen their operational research capacity to evaluate supervision approaches.

To implement effective HCW supervision, we recommend national and subnational stakeholders seek to contextualize, integrate, and optimize the following supervision enhancements within health systems:

*Evidence-based, QI tools and processes.* QI methods streamlining HCW performance management data with other health system performance data and information flows can help HCW supervisors to effectively assess quality gaps, address underlying factors, and continuously monitor and adapt through collaborative problem-solving, measurement, and data use. Clinical mentoring can effectively complement routine supervision, particularly for task shifting/task sharing. Constructive feedback should be timely and include positive recognition to motivate health workers.

*Digital supervision data and supervision processes.* Using digital checklists or job aids with algorithms can facilitate adherence to standards and deliver the most appropriate and immediate feedback. This is critical for supervising health workers who serve in remote locations and/or those working in emergency contexts, such as a pandemic, civil strife, or natural disaster. In health systems with reliable access to basic hardware and software, electricity, and connectivity, collecting and disseminating supervision data digitally can support potential advantages and efficiencies for the supervisor, including reducing paper-based data management tasks and automating analysis to demonstrate performance trends or target supervisee support needs. Digital data—including patient-level data—generated by remote or community-based HCWs, helps supervisors follow supervisees’ activities, monitor quality, and improve feedback loops [36].

*Improved interoperability and use of national HMIS.* Reviewing facility and service area performance and prioritizing supervisory support to the lowest performing areas can help target resource allocation and improve quality and equity. Integrating supervision systems’ information into the same platforms that host other health systems data (e.g., DHIS2), may offer more opportunities to provide real-time information and feedback to the supervisor and supervisee to promote evidence-based problem solving. Integrating supervision data with an HMIS could promote efficiencies and possibly cost-effectiveness for HCW performance and health system management processes. Integrating or linking health worker performance data to health systems outputs, including the quantity and quality of specific services in comparison with data on the population of district-level dashboards [19], can be especially useful when ensuring the effectiveness of community-to-facility referral systems [21].

To sustain effective supervision, we recommend to:

*Scale and replicate successful models across service delivery areas and geographies*. Promising supervision systems can be adapted to expand use for the public and private sector, and for facility- and community-based workers. Supervision approaches should not create a vertical system competing with other national or district level activities, but rather demonstrate adaptability to multiple programs, and facilitate targeted supervision within an integrated context in response to an assessment of facility-wide performance.

*Expand and institutionalize supervision to reach, prepare, protect, and support frontline health workers*. The COVID-19 pandemic has placed additional physical, biological, psychosocial and occupational risks on health workers and compounds the challenges they face to deliver high-quality care, especially as they adapt and respond to an emerging threat [64]. Traditional methods of routine supervision have been interrupted or curtailed due to new facility regulations, physical distancing requirements, and reduced mobility and time for supervisors and program managers to dedicate to mentoring and training, including to apply new COVID-19 and infection prevention and control protocols. Institutionalizing supervision is important to prepare for emergencies and establish support mechanisms for frontline health worker. Supervision enhancements—including using digital platforms, communicating by phone/text, conducting remote training, and using digital checklists—can be meaningful if in-person visits are not possible to fill the gaps, when HCWs need more support than ever.

*Transition and sustain efforts with local human and financial resources.* Community engagement and feedback on the quality of services can complement district- or manager-level supervisory efforts, especially for supervising community health workers as they are increasingly professionalized [65]. Delegating supervisory roles to both facility- and community-based workers can increase the number of supervisory contacts and improve accountability, especially when introducing or scaling a new CHW task or when facility-based supervisors face high workloads. A whole of system approach may help to address the HCW enabling environment more locally and sustainably. Supervision approaches should engage a range of stakeholders across local systems, including district managers, peers, and communities. For those who least benefit from routine supervision, such as CHWs, programs can utilize interprofessional or group assessments. These approaches can promote efficiencies, where site or field visits consume supervisor time and resources are not possible due to travel restrictions.

## Conclusion

Effective supervision enhancements and innovations should be better integrated, scaled, and sustained within existing systems to improve access to quality health care. Enhanced health worker supervision can better increase HCW performance and strengthen health systems when it is informed by and promotes continual, routine use of supervisory and health system information using a QI-focused modality. Sharing health worker supervision data through digital platforms to deliver immediate feedback loops for the supervisees, health systems actors, and local communities promotes greater awareness of performance issues to foster better awareness, accountability, and action.

Sustaining successful supervision approaches requires adequate human and financial resources, integrating visits into other health system activities, and adapting beyond a single health worker type or disease area. Enhancements are too often one-off, donor-funded approaches that are program-driven rather than country-led, whole-system changes that can be scaled up and sustained over time. Further country-led research is recommended to assess the outputs, outcomes, effects, and impact of the same enhanced supervision approach across different country contexts and health areas, for different types of health workers (e.g., public and private sector, facility- and community-based), and at different stages of maturity.

## Supplementary Information

**Additional file 1.** Supervision review inventory and taxonomy lists.

## Data Availability

The data sets generated or analyzed during this study are included within this article and its Additional file.

## Abbreviations

CASP: Critical Appraisal Skills Program
CHW: Community health worker
HRH: Human resources for health
HCW: Health care worker
HRH2030: Human Resources for Health in 2030 Program
LMIC: Low- and middle-income country
NGO: Non-governmental organization
QI: Quality improvement
USAID: United States Agency for International Development
